# Cyclic AMP Rescue of Motility in Sperm Devoid of Soluble Adenylyl Cyclase

**DOI:** 10.3390/ijms26041489

**Published:** 2025-02-11

**Authors:** Sylvia Ayoub, Natalia del R. Rivera Sanchez, Justine Fischoeder, Melanie Balbach, Lonny R. Levin, Jochen Buck, Carla Ritagliati

**Affiliations:** Department of Pharmacology, Weill Cornell Medicine, New York, NY 10065, USAllevin@med.cornell.edu (L.R.L.);

**Keywords:** cyclic AMP (cAMP), sperm motility, soluble adenylyl cyclase (sAC)

## Abstract

The second messenger cAMP plays multiple critical roles in the control of sperm functions essential for male fertility, including motility. The enzyme soluble adenylyl cyclase (sAC; ADCY10) was shown genetically and pharmacologically to be the essential source of cAMP mediating many of these functions. Male mice and men with genetic deletions of sAC are infertile, and their sperm are progressively immotile. Pharmacologically, delivery of potent and specific sAC inhibitors to male mice renders them temporarily infertile, and their sperm are similarly immotile. Here, we show that males from a second, independently derived mouse sAC knockout line are also infertile with progressively immotile sperm. We use these mouse models to determine optimal conditions for pharmacologically elevating intracellular cAMP to rescue the sAC null motility defect. We show that cell-permeable cAMP analogs, but not forskolin, rescue the motility defects of sAC deficient sperm, and we demonstrate that 8Br-cAMP is an efficient cAMP analog to rescue motility.

## 1. Introduction

Cyclic AMP (cAMP) was the first second messenger signaling molecule identified. Shortly after its discovery, Garbers and co-workers explored whether it may play a role in sperm biology. They found that treatments that elevate intracellular cAMP, either by inhibiting the phosphodiesterases that break down cAMP or by addition of the membrane-permeable cAMP analog, dibutyryl cAMP, increased sperm motility [1,2,3]. Since those initial discoveries, the relationship between cAMP and mammalian sperm motility has been demonstrated in every mammal tested (including humans) [4,5,6].

Intracellular cAMP signaling is controlled via the balance between its synthesis by adenylyl cyclases (ACs, catalyzing the conversion of ATP into cAMP) and degradation by phosphodiesterases (PDEs). In mammals, there are two different types of ACs: transmembrane ACs (tmACs) and soluble AC (sAC). TmACs mediate cAMP-dependent responses to hormones and neurotransmitters. They are regulated by heterotrimeric G proteins and stimulated by the pharmacological tool forskolin [7]. In contrast, sAC is insensitive to heterotrimeric G proteins and forskolin [8]. In most mammals, nine genes encode different tmAC isoforms (ADCY1–9), displaying unique tissue and cell-type specific expression patterns and different modes of regulation. G protein-mediated, hormone-dependent [9,10] and forskolin-responsive [11] cAMP synthesizing activities have been identified in sperm. From gene knockout studies of the nine mouse tmAC genes, only ADCY3 KO mice display male-specific subfertility [12], and sperm from these mice exhibit reduced fertility in in vitro fertilization, thought to be due to a deficiency in acrosome reaction.

In contrast to the tmAC subfamily, mammals possess a single gene encoding soluble adenylyl cyclase (sAC; ADCY10) [8], which generates multiple isoforms via alternative splicing. Also distinct from tmACs, sAC activity is directly stimulated by HCO_3_^−^ and Ca^2+^ [13,14,15,16,17]. sAC is most abundantly expressed in testes and sperm [8,18,19], and its essential role in male reproduction was confirmed both genetically [20,21,22,23,24] and pharmacologically [21,23,25,26,27,28] in mice and men.

After being produced in the testes, mammalian sperm are stored in the cauda region of the epididymis, where they are morphologically mature but functionally immature, unable to fertilize an egg. Upon ejaculation, sperm encounter high concentrations of HCO_3_^−^ and Ca^2+^ [29,30], initiating motility and a post-ejaculation maturation process termed capacitation [31,32]. Multiple events occurring during capacitation, including activation of motility and changes in the motility pattern, are dependent upon sAC-produced cAMP (reviewed in [5,6]). The main signaling pathways controlling the motility of mammalian sperm are regulated by Ca^2+^ and cAMP, and sAC integrates both: incubation with HCO_3_^−^ and Ca^2+^ stimulates sAC activity, which produces cAMP, increasing the flagellar beat frequency. In vivo, motility activation occurs when sperm are released from the epididymis and encounter reproductive fluids in the male and/or female genital tract, containing high HCO_3_^−^ levels [33].

Alternative splicing generates at least two transcripts from the ADCY10 locus: full-length sAC (sACfl), encoding a 187 kDa protein, and truncated sAC (sACt), encoding a 48 kDa protein [8,34]. Both sAC protein isoforms can be found in mouse testes [21]. sACt is comprised almost exclusively of two homologous catalytic domains (C1 and C2) [8,34]; it lacks the regulatory domains present in sACfl and exhibits significantly higher specific activity [35]. Subsequent cloning experiments identified a second start site, which predicts that there are sAC isoforms missing the first catalytic domain [18]. However, these predicted sAC C2-only isoforms have never been biochemically characterized, and all work describing sAC physiological regulators and pharmacological inhibitors refers to the sAC C1–C2 isoforms abundantly expressed in testes and sperm [27].

Gene knockout studies removing exons encoding the C1 domain confirm that known sACt and sACfl isoforms are essential for sperm to fertilize an egg. sAC C1 knockout (KO) mice show male-specific sterility; their sperm are immotile, unable to fertilize an oocyte in vitro, and fail to reach the fertilization site in vivo [20,21,24,36]. The motility defect of sperm from sAC C1 KO mice can be rescued by the addition of exogenous, membrane-permeable cAMP analogs, confirming that their motility defect is due to loss of sAC and sAC-generated cAMP. A second ubiquitous KO mouse model, selectively removing the second catalytic domain (C2), was also reported to be male-specific sterile [37]; however, these mice (sAC C2 KO) were never further characterized. In humans, two sAC null men were identified to be infertile. Like sperm from sAC C1 KO mice, sperm from these infertile men were immotile, and motility was restored by the addition of cell-permeable cAMP [22]. More recently, additional infertile men have been identified with mutations abrogating sAC activity [38].

The aim of this work was to determine whether sperm from sAC C2 KO mice exhibit similar motility defects as the sperm from sAC C1 KO mice and to explore the requirements to rescue the motility defects of sAC-deficient sperm.

## 2. Results

**Figure 1:**
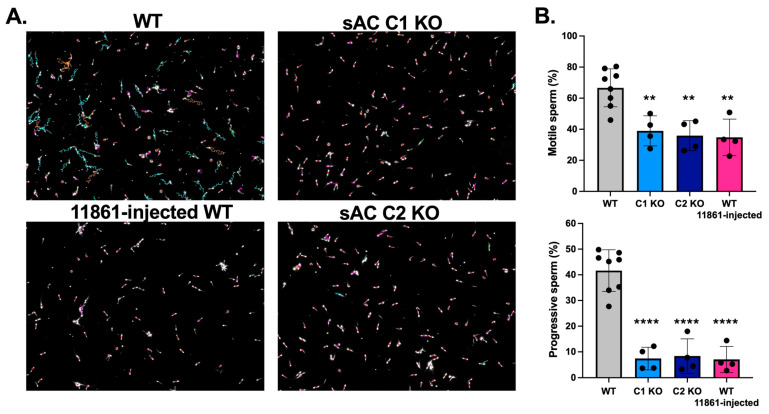
Genetic and pharmacological disruption of sAC activity renders sperm immotile. Motility of epididymal sperm from WT (gray), sAC C1 KO (light blue), sAC C2 KO (dark blue), and 11861-injected WT males (pink). (**A**) Representative images with motility tracks of the CASA obtained with IVOSII Hamilton Thorne. Track’s color code: motile (green), progressive (turquoise), hyperactivated (orange), slow (pink), static (red). (**B**) Bar graphs show percentage total (progressive + slow or twitching sperm) and progressive motility. Data are shown as means ± SEM, representative of at least four independent experiments, with the individual values for each experiment indicated. More than 500 sperm in at least five fields were analyzed. One-way ANOVA was performed, with Tukey’s multiple comparisons test. ** *p* < 0.005, **** *p* < 0.0001 statistical significance of each condition with the WT. No significant differences between the other conditions.

While sAC C1 KO mice and sperm have been extensively analyzed, this is the first study characterizing the mechanism of male infertility in sAC C2 KO mice. Similar to sAC C1 KO, and in agreement with [37], sAC C2 KO mice did not exhibit developmental abnormalities, and the males were sterile. Five sAC C2 KO males were harem-mated with WT females for four days; after this time, the females were replaced by new ones, for a total of three rounds for each male. No litters were obtained. We analyzed the motility of sperm extracted from the cauda epididymis using Computer-Assisted Sperm Analysis (CASA). As the representative videos show, while WT sperm were rapidly and progressively motile, sperm from sAC C2 KO were immotile, barely twitching, resembling the phenotype of sperm from sAC C1 KO males (Movie S1). As previously described, similar motility defects were seen in sperm from WT males injected with the sAC inhibitor TDI-11861 [28]. In some cases, the CASA detects the vibratory or twitching movement as slowly motile, but progressively immotile, meaning they just twitch in place (Figure 1). Thus, sperm from mice devoid of active sAC, whether from genetic ablation of the C1 catalytic domain or the C2 catalytic domain or when pre-treated with a potent sAC inhibitor, are effectively immotile.

**Figure 2:**
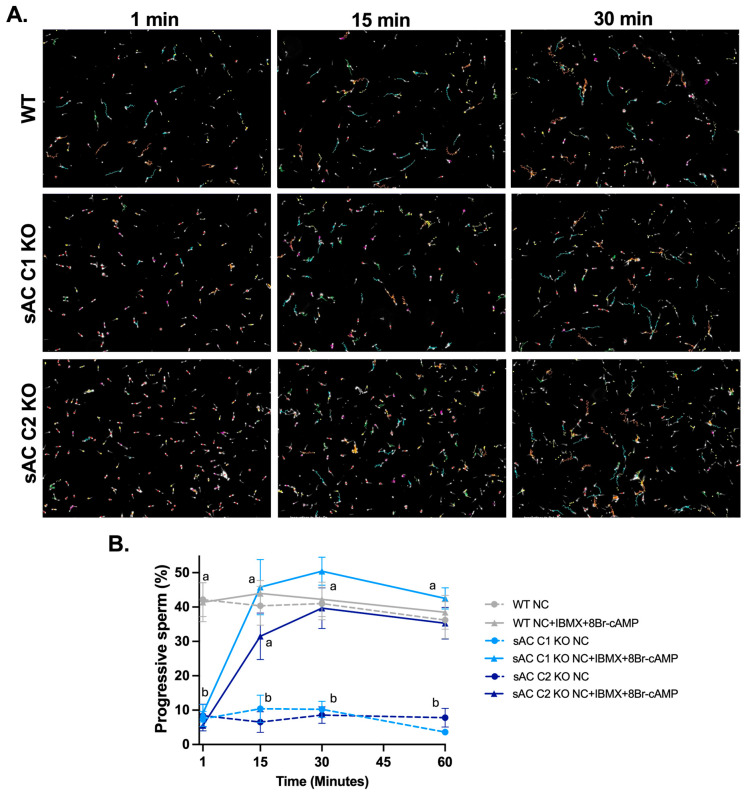
Kinetic of motility recovery of sAC-deficient sperm with an external cAMP analog. Epididymal WT (gray), sAC C1 KO (light blue), and sAC C2 KO (dark blue) sperm incubated in the absence (dotted lines) or presence (full lines) of 1 mM 8Br-cAMP and 0.5 mM IBMX and analyzed in the CASA at different time points. (**A**) Representative images with motility tracks obtained with IVOSII Hamilton Thorne. Track’s color code: motile (green), progressive (turquoise), hyperactivated (orange), slow (pink), static (red). (**B**) Bar graphs show percentage of progressive motility. Data are shown as means ± SEM, representative of four independent experiments. Two-way ANOVA was performed, with Tukey’s multiple comparisons test. Different letters indicate statistically significant differences between the conditions within each time point. More than 500 sperm in at least five fields were analyzed.

To confirm that their motility defects are specifically due to the absence of sAC and sAC-generated cAMP, we analyzed the ability of membrane-permeable cAMP analogs to restore motility of sAC C1 KO and sAC C2 KO sperm. First, we explored the time it took for the membrane-permeable cAMP analog, 8Br-cAMP, in the presence of the broad specificity phosphodiesterase inhibitor IBMX, to restore motility to sAC C1 KO and sAC C2 KO sperm. As expected, in the absence of external cAMP, epididymal sAC C1 KO and C2 KO sperm exhibited no progressive motility. After 30 min in the presence of 8Br-cAMP and IBMX, sperm from both sAC C1 KO and sAC C2 KO mice were recovered to the same extent as observed for WT sperm (Figure 2 and Figure S1, and Movie S2).

**Figure 3:**
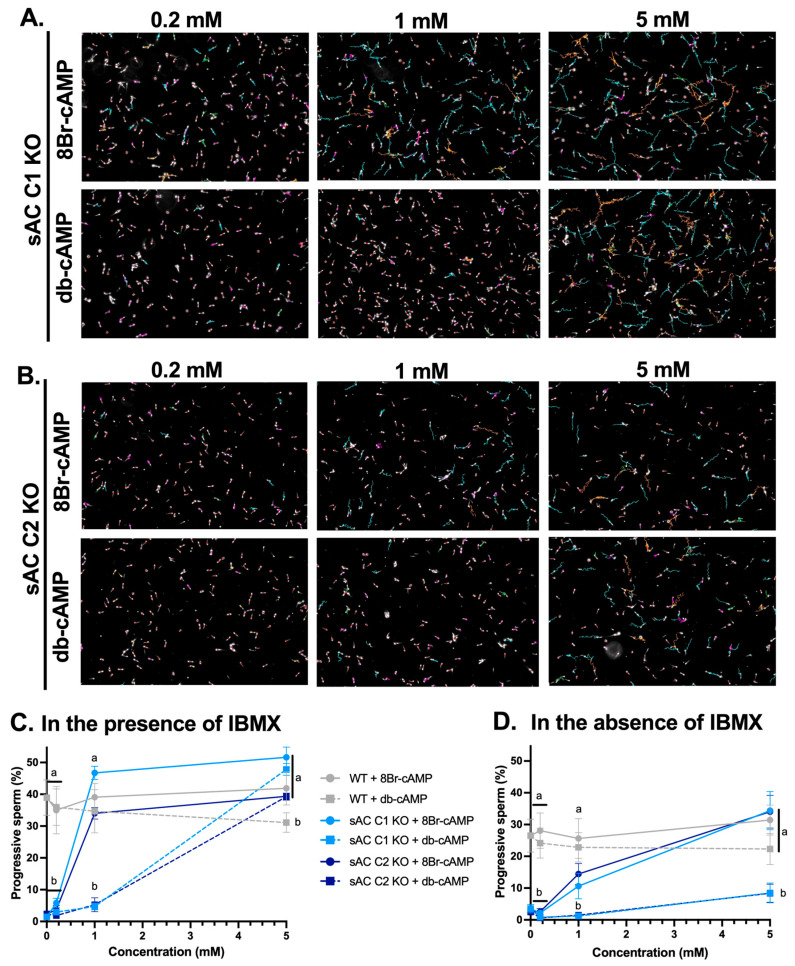
Recovery of motility of sperm from sAC KO mice with increasing concentrations of two different cAMP analogs. Epididymal WT (gray), sAC C1 KO (light blue), and sAC C2 KO (dark blue) sperm incubated with increasing concentrations of 8Br-cAMP or db-cAMP, in the presence (**A**–**C**) or absence (**D**) of 0.5 mM IBMX. (**A**,**B**) Representative images with motility tracks obtained with IVOSII Hamilton Thorne CASA. Track’s color code: motile (green), progressive (turquoise), hyperactivated (orange), slow (pink), static (red). (**C**,**D**) Bar graphs show percent progressive motility. Data are shown as means ± SEM. Two-way ANOVA was performed, with Tukey’s multiple comparisons test. Different letters indicate statistically significant differences between the conditions within each time point. More than 500 sperm in at least five fields were analyzed.

We next compared the efficacies of the two most widely used cAMP analogs. While both are membrane-permeable cAMP analogs, which will elevate intracellular cAMP levels, upon entering cells, db-cAMP is metabolized into native cAMP, which is subject to degradation by phosphodiesterases. db-cAMP also carries a butyrate conjugate. In contrast, when 8Br-cAMP enters cells, it is less sensitive to phosphodiesterase cleavage, implying that it would result in more sustained elevated intracellular levels of the second messenger. We examined the efficacy of each cAMP analog to restore sperm motility from both sAC KO lines after 30 min in the absence (Supplemental Figure S2 and Movie S3) and presence (Figure 3 and Movie S4) of IBMX. With both cAMP analogs, motility was dose-dependently recovered in sperm from both sAC KO lines. However, as predicted from its greater intracellular stability, 8Br-cAMP rescued motility substantially better than db-cAMP, especially in the absence of IBMX (Supplemental Figure S2, Movies S3 and S4, and Figure 3). Without the addition of IBMX, even the highest db-cAMP concentration tested was not sufficient to support full recovery (Movie S3, Supplemental Figure S2, and Figure 3D).

Initial studies exploring molecular hallmarks associated with capacitation (i.e., protein tyrosine phosphorylation) revealed that cAMP mediated the effects of bicarbonate independent of BSA [39]; therefore, we tested whether BSA impacts the ability of cAMP to regulate motility. The results observed in previous experiments (Figure S2 and Figure 3), which were performed in the absence of BSA, were unaffected when BSA was added to the incubation media; inclusion of BSA did not shift or alter the dose responses to recover sperm motility by the different cAMP analogs (Supplemental Figure S3).

**Figure 4:**
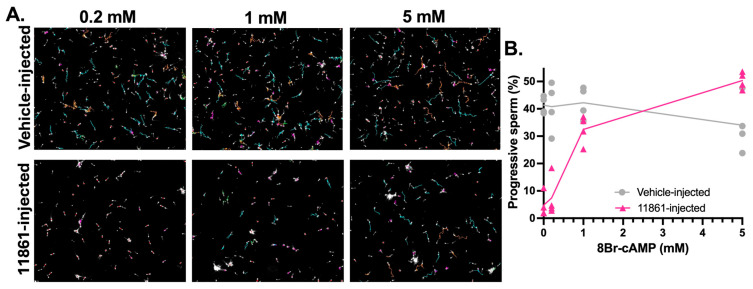
Recovery of motility of sperm from sAC-inhibited mice with increasing concentrations of 8Br-cAMP. Epididymal WT sperm from vehicle-injected (gray) or 11861-injected (pink) males, incubated with increasing concentrations of 8Br-cAMP in the presence of 0.5 mM IBMX. (**A**) Representative images with motility tracks of the CASA obtained with IVOSII Hamilton Thorne. Track’s color code: motile (green), progressive (turquoise), hyperactivated (orange), slow (pink), static (red). (**B**) Bar graphs show percent progressive motility. Data are shown as means ± SEM, representative of four independent experiments, with the individual values for each experiment indicated. More than 500 sperm in at least five fields were analyzed.

Additionally, to explore whether sperm from mice with sAC genetically ablated harbored any chronic defects or compensatory adaptations, we assessed if the ability of exogenous cAMP to rescue sperm motility differed in sperm where sAC activity was pharmacologically blocked. The motility of epididymal sperm surgically extracted from TDI-11861-injected males was analyzed after 30 min in the presence of increasing concentrations of 8Br-cAMP. The motility of sperm where sAC was pharmacologically inhibited was dose-dependently recovered at similar concentrations (Figure 4 and Movie S5) to sAC C1 KO and sAC C2 KO sperm.

**Figure 5:**
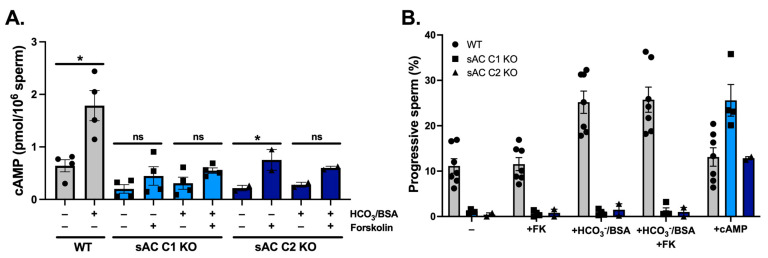
sAC is the sole source of cAMP capable of supporting sperm motility. Epididymal WT (circles, gray), sAC C1 KO (squares, light blue), and sAC C2 KO (triangles, dark blue) sperm were incubated in non-capacitating (−)or capacitating (HCO_3_^−^/BSA) conditions, in the absence or presence of 50 μM forskolin (FK) or with 1 mM 8Br-cAMP and 0.5 mM IBMX (cAMP). (**A**) Intracellular cAMP after incubation for 15 min in the presence of 0.5 mM IBMX. Individual replicates indicated by symbols. Ratio paired Student’s *t* test was performed between indicated conditions: * *p* < 0.05, ns (non-significant). (**B**) Bar graphs show progressive motility. Data are shown as means ± SEM, with the individual values for each experiment indicated. More than 500 sperm in at least five fields were analyzed.

Finally, we asked whether sAC was the sole endogenous source of cAMP in sperm capable of supporting motility. We first observed that there is cAMP in sAC KO sperm, confirming that an alternate source of cAMP, presumably tmACs, is present in sperm (Figure 5A). Previous studies leveraging hormones that traditionally signal via G protein-dependent stimulation of tmACs (i.e., adenosine and catecholamine agonists) showed that these hormones modulate sperm motility via sAC and not via tmACs [40], suggesting that tmAC-generated cAMP may be unable to modulate sperm motility. We took a more general approach to test the involvement of tmACs in sperm motility by using forskolin, which is a non-specific stimulator of the tmAC subfamily [7]. Forskolin elicited a small elevation of intracellular cAMP, which was significant in sAC C2 KO sperm but not in sAC C1 KO sperm (Figure 5A). However, addition of forskolin did not alter the motility of WT sperm; nor did it rescue the immotility of the sAC KOs sperm (Figure 5B). As controls, motility of WT sperm remained normally responsive to sAC-dependent elevations of cAMP due to addition of HCO_3_^−^/BSA, and sAC KO sperm were rescued by addition of exogenous 8Br-cAMP.

## 3. Discussion

Initial biochemical studies identifying a soluble enzyme containing adenylyl cyclase activity only detected activity in testis cytosol [41,42]. Upon cloning the single ADCY10 gene encoding sAC, northern blot data showed that sAC mRNA is only abundantly expressed in testes, specifically enriched within developing male germ cells [8,43]. However, the ADCY10 locus is complex, generating multiple isoforms via alternative splicing [8,34] and alternate promoter utilization [18,44]. The original isolation of sAC from rat testes identified cDNAs encoding two protein isoforms; a full-length isoform (187 kDa) and a truncated isoform (48 kDA) comprised of two related cyclase catalytic domains [8,19,34]. Both isoforms are found in testes [21]. Additional alternatively spliced variants of sAC were subsequently reported from different tissues outside the testis [18,19,43,45], and genetic and pharmacological experiments identified sAC functions beyond male-specific sterility (Reviewed in [46]). A somatic-specific sAC protein containing only the second of its two essential catalytic domains is predicted to derive from mRNAs transcribed from a different promoter [18,27,44]. In an attempt to separately study these predicted C2-only sAC isoforms, we generated a second sAC KO mouse model (C2 KO), selectively removing three exons encoding the second catalytic domain [37]. Similar to sAC C1 KO mice, sAC C2 KO mice were male-specific sterile with no other obvious phenotypes. Because the fertility defect of sAC C2 KO mice was never characterized further, we explored C2 KO sperm and compared them directly with sAC C1 KO sperm. We now show that sAC C2 KO mice did not exhibit developmental abnormalities, and the males were sterile, despite showing normal mating behavior, like sAC C1 KO, and in agreement with [37]. Epididymal sperm were morphologically normal, but immotile, resembling the motility defects observed in sperm from sAC C1 KO and from WT mice injected with a sAC inhibitor.

To confirm that their motility defects are specifically due to the absence of sAC activity and sAC-generated cAMP, we compared the ability of the two most widely used membrane-permeable cAMP analogs (8Br-cAMP and db-cAMP) to restore the motility of sperm from sAC C1 KO, sAC C2 KO, and TDI-11861-injected WT males. In all cases, both cAMP analogs dose-dependently restored sperm motility. However, as predicted from its greater intracellular stability, 8Br-cAMP was more potent than db-cAMP, especially in the absence of IBMX. These experiments provide a method to exogenously supply cAMP when sAC activity is abrogated.

Lastly, we assessed the ability of tmAC-produced cAMP to rescue the sAC-deficient motility defect. If there is an increase in cAMP with forskolin, it is at the limit of detection, and, more importantly, it is irrelevant for motility. This might be because the levels of cAMP produced are not sufficient or because tmAC-generated cAMP is located in a different subcellular compartment not relevant for sperm motility.

In this study, we focused on the source of cAMP mediating sperm motility; we did not address which of the many cAMP effector cascades are relevant for motility. Effectors of cAMP include protein kinase A (PKA), exchange protein activated by cAMP (EPAC), different types of cAMP regulated channels, and the still poorly characterized family of Popeye domain containing proteins (POPDC). The membrane-permeable cAMP analogs used in this study do not distinguish among these different effectors. Future studies leveraging effector-selective cAMP analogs can explore which cAMP effector cascades mediate the different types of cAMP-regulated sperm motility.

## 4. Materials and Methods

### 4.1. Animals

The transgenic C1 sAC null mouse line, in which exons 2–4 are deleted, was generated at Lexicon Genetics (The Woodlands, TX, USA). For the transgenic C2 sAC null mouse cell line, a genomic 2.2 kb region comprising exons 11 to 13 (encoding much of the C2 catalytic domain) of mouse Adcy10 was flanked by loxP sites and subsequently removed via crossing to C57Bl/6 mice expressing Cre recombinase to generate a germline deletion of the C2 domain of Adcy10 [37]. Animal experiments were approved by Weill Cornell Medicine’s Institutional Animal Care and Use Committee (IACUC).

### 4.2. Reagents

3-Isobutyl-1-methylxanthine (IBMX), bovine serum albumin (BSA), NaHCO_3_, DMSO, and dibutyryl-cAMP were purchased from Sigma-Aldrich (St. Louis, MO, USA). 8-bromo-cAMP (sodium salt) came from TOCRIS (Minneapolis, MN, USA).

### 4.3. Sperm Preparation

Mouse sperm were isolated by incision of the cauda epididymis of male mice, followed by a swim-out in 500 μL Toyoda Yokoyama Hoshi (TYH) medium (in mM: 119.3 NaCl, 4.7 KCl, 1.7 CaCl_2_, 1.2 KH_2_PO_4_, 1.2 MgSO_4_, 5.6 glucose, 0.5 pyruvate, 20 HEPES, pH 7.4 adjusted with NaOH), prewarmed at 37 °C and counted using a hematocytometer.

### 4.4. Sperm Motility Analysis

Sperm suspensions were loaded on 100 μm deep Leja slides and placed on a microscope stage at 37 °C. Sperm movements were examined using an IVOSII Hamilton Thorne. Parameters used were as follows: 45 frames acquired, frame rate of 60 Hz, and cell size of 25–200 μm^2^. At least 5 microscopy fields and a minimum of 500 sperm were analyzed in each experiment. The following parameters were measured: mean path velocity (VAP, μm/s), curvilinear velocity (VCL, μm/s), straight-line velocity (VSL, μm/s), linearity (LIN, %), amplitude of lateral head displacement (ALH, μm), beat cross frequency (BCF, Hz) and straightness (STR, %). Sperm were considered hyperactivated when presenting VCL ≥ 271 μm/s, LIN < 50%, and ALH ≥ 3.5 μm and progressive when presenting VAP ≥ 50 μm/s and STR > 80%.

### 4.5. Sperm cAMP Quantification

Aliquots of 2–3 × 10^6^ WT or sAC C1 KO sperm were incubated for 15 min in the presence of 0.5 μM IBMX in non-capacitating (TYH) or capacitating (TYH + HCO_3_/BSA) media. Sperm were sedimented by centrifugation at 2000× *g* for 3 min and lysed in 200 μL of 0.1 M HCl for 10 min. Sperm lysates were centrifuged at 2000× *g* for 3 min, and the cAMP in the supernatant was acetylated and quantified using the Direct cAMP ELISA Kit (ENZO, catalog no. ADI-901-066A) according to the manufacturer’s instructions.

## Data Availability

The original contributions presented in this study are included in the article/Supplementary Materials. Further inquiries can be directed to the corresponding authors.

## Supplementary Materials

The following supporting information can be downloaded at https://www.mdpi.com/article/10.3390/ijms26041489/s1.
