# Supplementary material for: Cyclic AMP Rescue of Motility in Sperm Devoid of Soluble Adenylyl Cyclase
Source: Int J Mol Sci. 2025 Feb 11;26(4):1489. doi: 10.3390/ijms26041489 (PMC11855772; doi:10.3390/ijms26041489)
Supplement: Supplementary file 1 [file ijms-26-01489-s001.zip › ijms-3447120-supplement material.pdf]

TITLE: Cyclic AMP rescue of motility in sperm devoid of soluble adenylyl cyclase

AUTHORS: Sylvia Ayoub, Natalia del Rosario Rivera Sanchez, Justine Fischoeder, Melanie Balbach[1], Lonny R. Levin, Jochen Buck, Carla Ritagliati.

Supplementary material

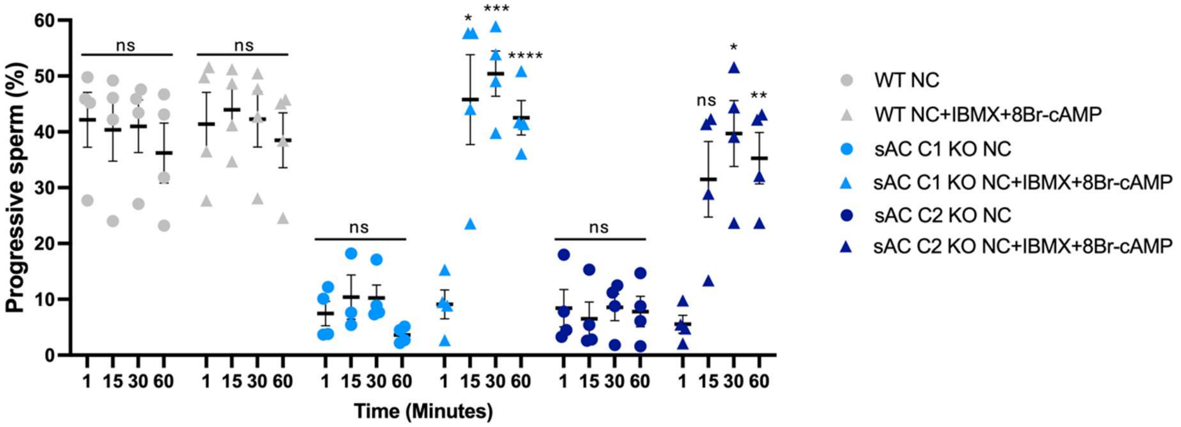

Supplemental Figure S1: Kinetic of motility recovery of sAC-deficient sperm with an external cAMP analog. Epididymal WT (grey), sAC C1 KO (light blue) and sAC C2 KO (dark blue) sperm incubated in the absence (circles) or presence (triangles) of 1 mM 8Br-cAMP and 0.5 mM IBMX and analyzed in the CASA at different time points. Bar graphs show percent progressive motility. Data are shown as mean ± SEM, representative of 4 independent experiments, with the individual values for each experiment indicated. More than 500 sperm in at least 5 fields were analyzed. Two-way ANOVA was performed, with Tukey's multiple comparisons test. *p < 0.05, **p < 0.005, ****p < 0.0001 statistical significance of each condition with its corresponding condition at 1 min. ns, No significant difference.

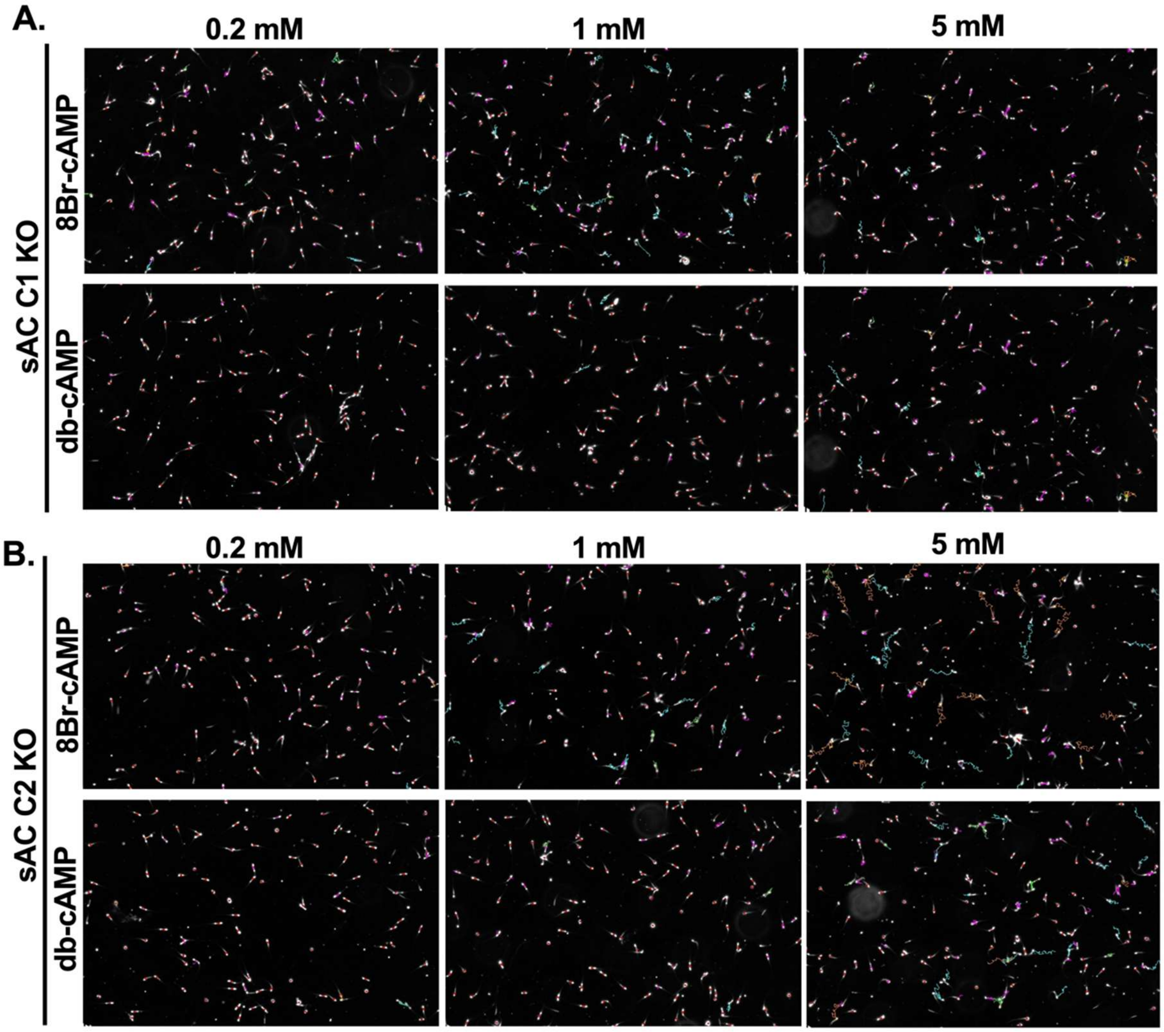

Supplemental Figure S2: Recovery of motility of sperm from sAC KO mice with increasing concentrations of two different cAMP analogs in the absence of IBMX. Representative images with motility tracks obtained with IVOSII Hamilton Thorne of epididymal WT, sAC C1 KO and sAC C2 KO sperm incubated with increasing concentrations of 8Br-cAMP or db-cAMP. Track´s color code: motile (green), progressive (turquoise), hyperactivated (orange), slow (pink), static (red).

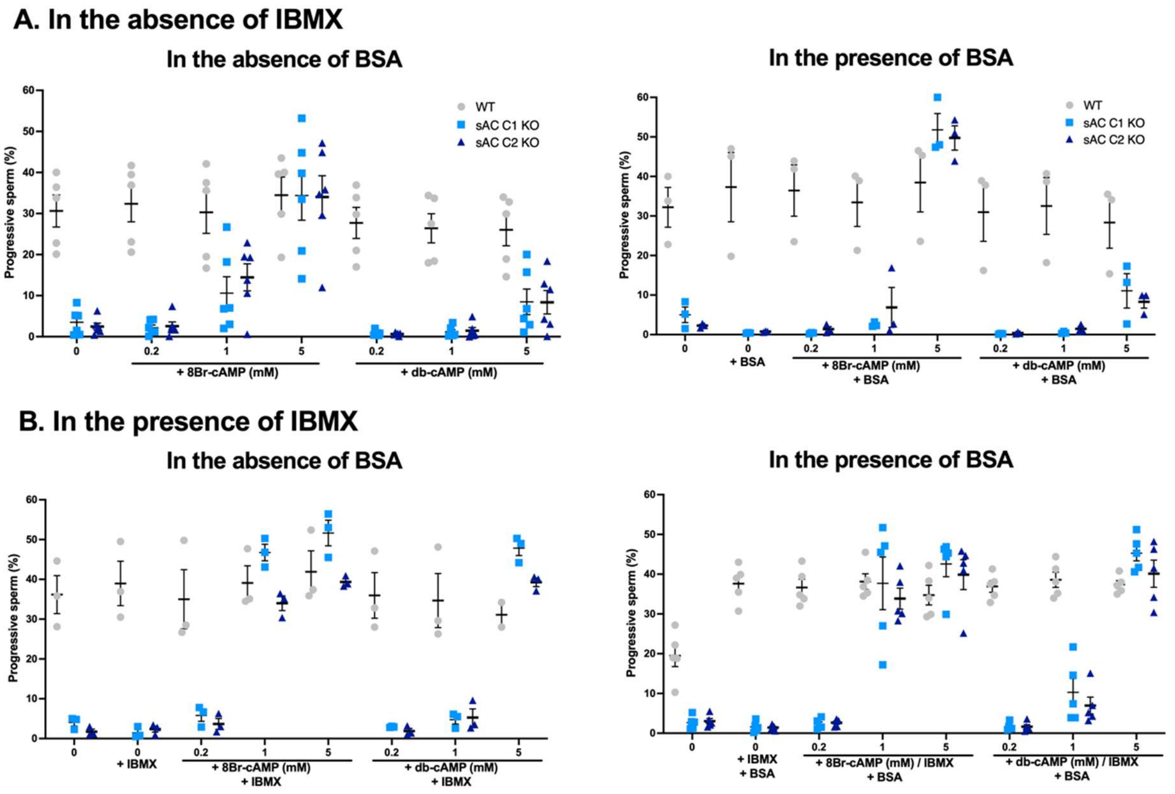

Supplemental Figure S3: BSA role in the cAMP recovery of sAC-deficient sperm motility. Epididymal WT (grey), sAC C1 KO (light blue) and sAC C2 KO (dark blue) sperm incubated with increasing concentrations of 8Br-cAMP or db-cAMP, in the absence (A) or presence (B) of 0.5 mM IBMX, without (left panels) of with (right panels) 5 mg/ml BSA. Data are shown as mean ± SEM, representative of at least 3 independent experiments, with the individual values for each experiment indicated. More than 500 sperm in at least 5 fields were analyzed.

Movie legends

Movie S1: Representative videos of epididymal sperm from WT, sAC C1 KO, sAC C2 KO and 11861-injected WT males obtained with IVOSII Hamilton Thorne. Motility track´s color code: motile (green), progressive (turquoise), hyperactivated (orange), slow (pink), static (red).

Movie S2: Representative videos of epididymal WT, sAC C1 KO and sAC C2 KO sperm incubated in the presence of 1 mM 8Br-cAMP and 0.5 mM IBMX and analyzed

in the CASA at different time points. Motility track´s color code: motile (green), progressive (turquoise), hyperactivated (orange), slow (pink), static (red).

Movie S3: Representative videos with motility tracks obtained with IVOSII Hamilton Thorne of epididymal WT, sAC C1 KO and sAC C2 KO sperm incubated with increasing concentrations of 8Br-cAMP or db-cAMP in the absence of IBMX. Track´s color code: motile (green), progressive (turquoise), hyperactivated (orange), slow (pink), static (red).

Movie S4: Representative videos with motility tracks obtained with IVOSII Hamilton Thorne of epididymal WT, sAC C1 KO and sAC C2 KO sperm incubated with increasing concentrations of 8Br-cAMP or db-cAMP in the presence of 0.5 mM IBMX. Track´s color code: motile (green), progressive (turquoise), hyperactivated (orange), slow (pink), static (red).

Movie S5: Representative videos with motility tracks obtained with IVOSII Hamilton Thorne of epididymal WT sperm from vehicle-injected or 11861-injected males, incubated with increasing concentrations of 8Br-cAMP in the presence of 0.5 mM IBMX.
