# Supplementary figures and images for: Cyclic AMP Rescue of Motility in Sperm Devoid of Soluble Adenylyl Cyclase
Source: Int J Mol Sci. 2025 Feb 11;26(4):1489. doi: 10.3390/ijms26041489 (PMC11855772; doi:10.3390/ijms26041489)

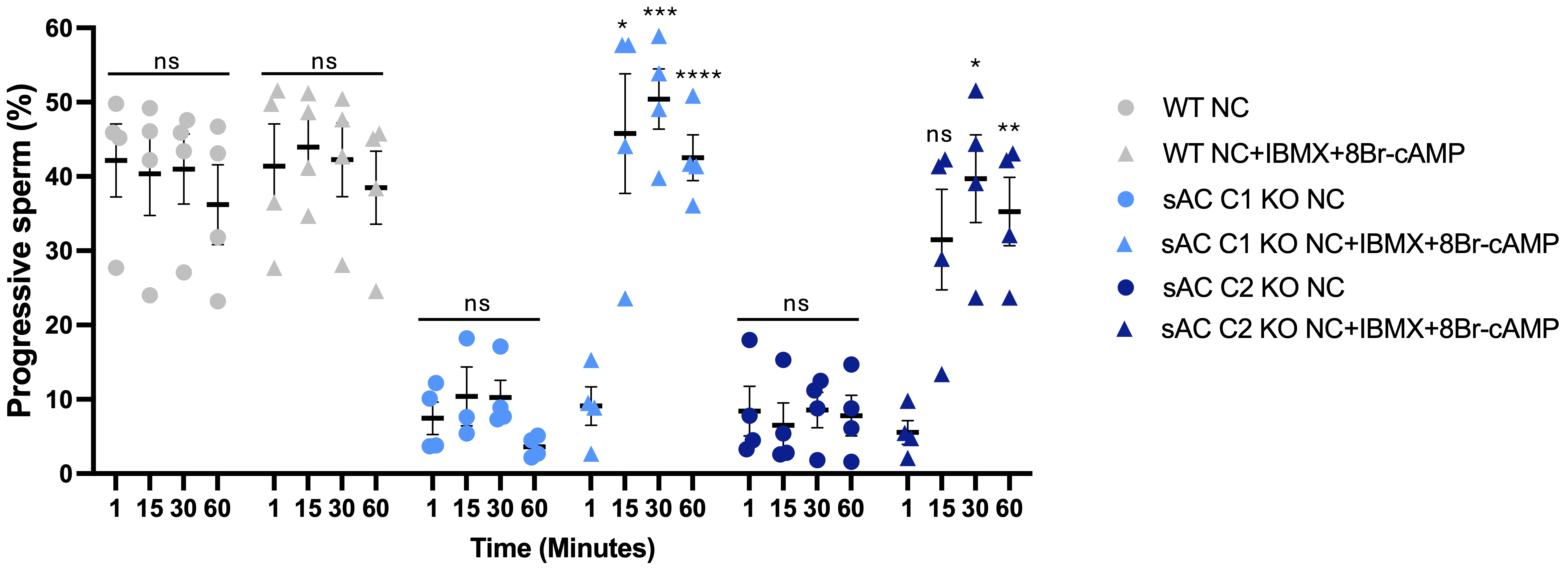

Supplement: Supplementary file 1 [file ijms-26-01489-s001.zip › S1.tiff]

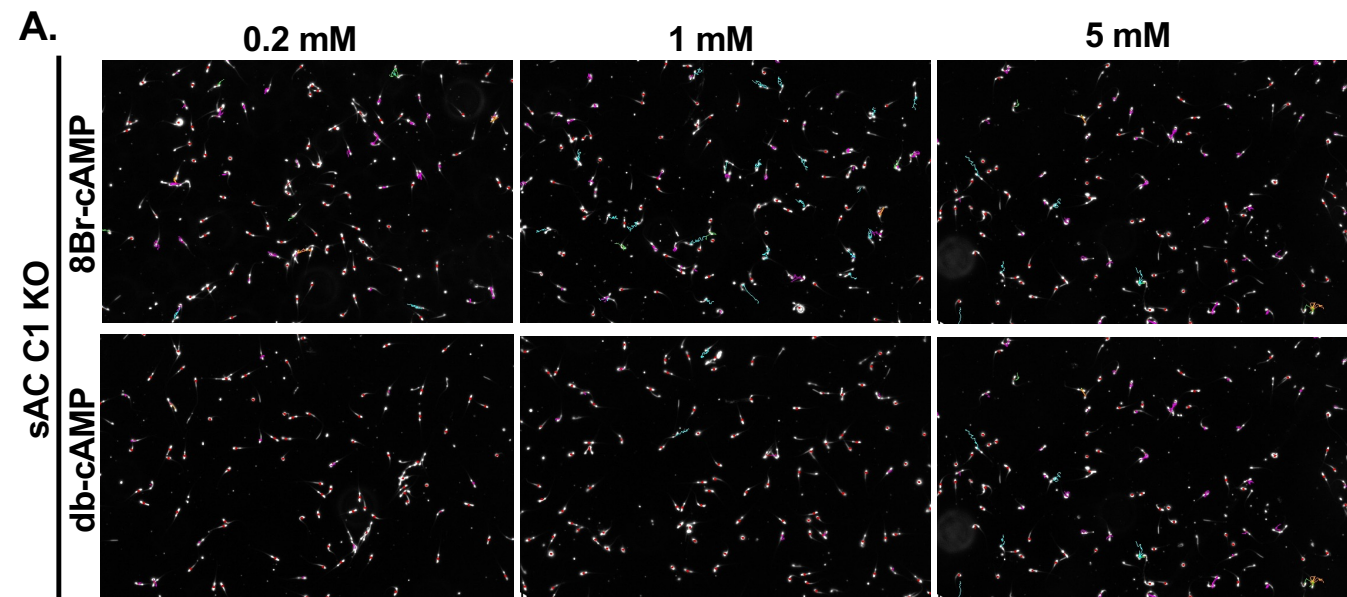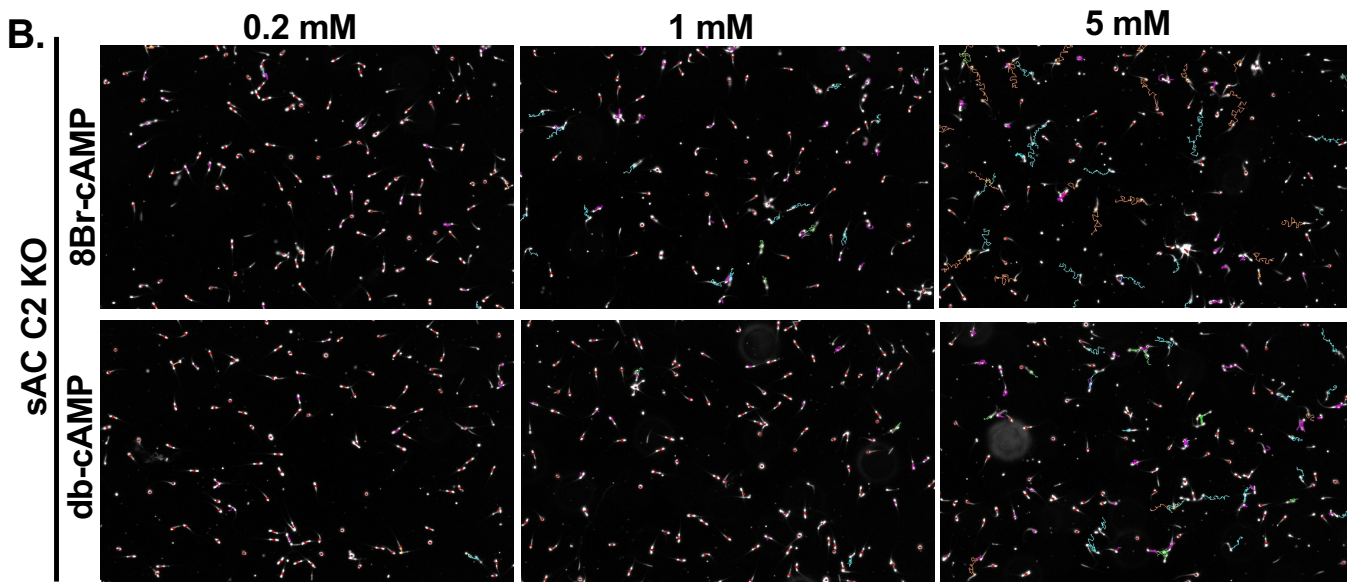

Supplement: Supplementary file 1 [file ijms-26-01489-s001.zip › S2.pdf]

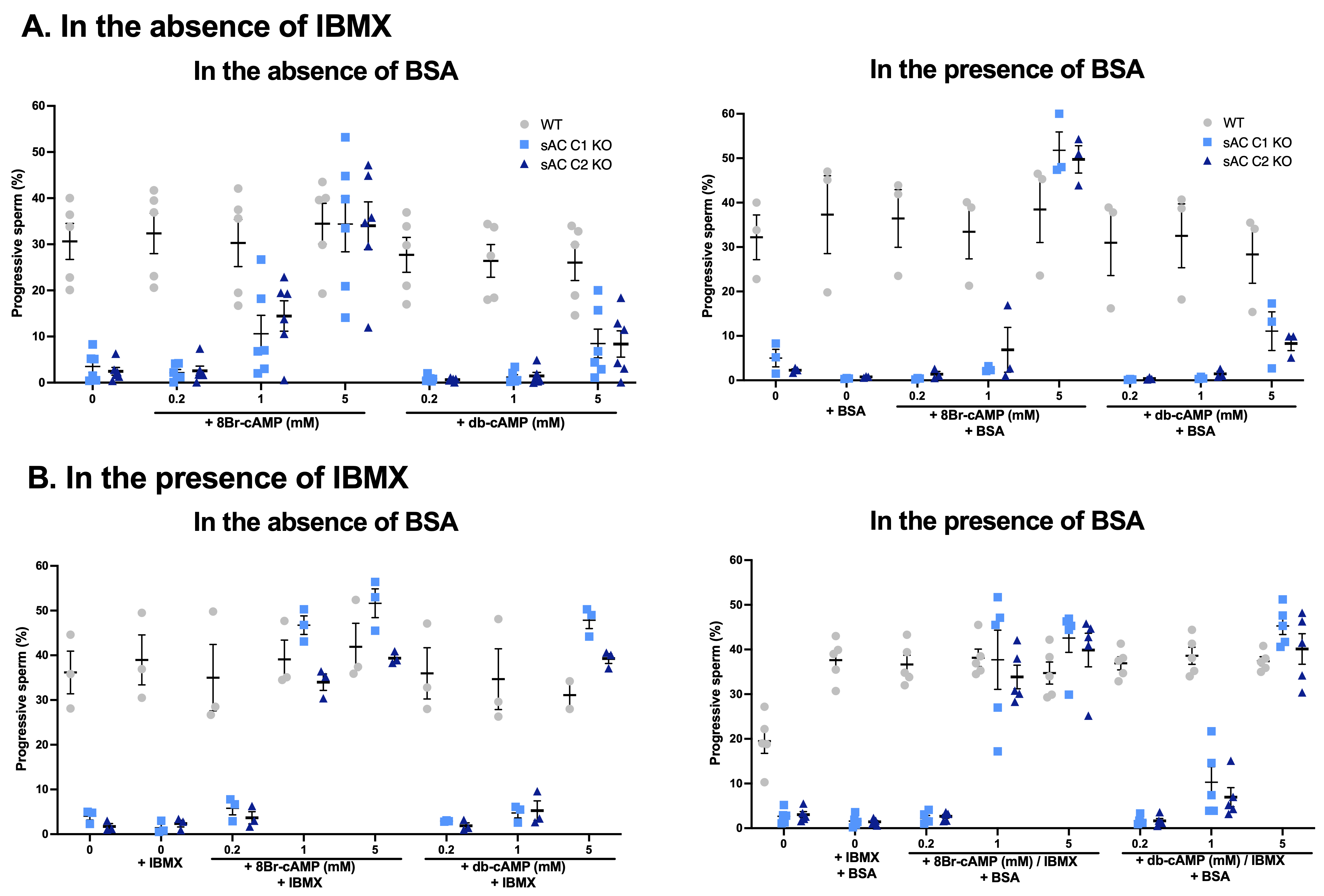

Supplement: Supplementary file 1 [file ijms-26-01489-s001.zip › S3.tiff]
